# Perceptions of the Targets and Sources of COVID-19 Threat are Structured by Group Memberships and Responses are Influenced by Identification with Humankind

**DOI:** 10.5334/pb.1043

**Published:** 2022-03-16

**Authors:** Svenja B. Frenzel, Nina M. Junker, Lorenzo Avanzi, Valerie A. Erkens, S. Alexander Haslam, Catherine Haslam, Jan A. Häusser, Daniel Knorr, Ines Meyer, Andreas Mojzisch, Lucas Monzani, Stephen D. Reicher, Sebastian C. Schuh, Niklas K. Steffens, Llewellyn E. van Zyl, Rolf van Dick

**Affiliations:** 1 Department of Social Psychology, Goethe University Frankfurt, Germany; 2 Department of Psychology, University of Oslo, Norway; 3 Department of Psychology and Cognitive Science, University of Trento, Italy; 4 Department of Social Psychology, Justus-Liebig-University Gießen, Germany; 5 School of Psychology, University of Queensland, Australia; 6 School of Management Studies, University of Cape Town, South Africa; 7 Psychology Department, University Hildesheim, Germany; 8 Ivey Business School, University of Western Ontario, Canada; 9 School of Psychology and Neuroscience, University of St Andrews, Scotland, UK; 10 China Europe International Business School (CEIBS), Shanghai China; 11 Eindhoven University of Technology, the Netherlands; 12 Human Performance Management, Optentia Research Focus Area, North-West University, South Africa; 13 Department of HRM, University of Twente, Netherlands

**Keywords:** threat perception, social groups, Social Identity Approach, psychological distance, COVID-19

## Abstract

The purpose of this study was to investigate which social groups are perceived as a threat target and which are perceived as a threat source during the COVID-19 outbreak. In a German sample (*N* = 1454) we examined perceptions of social groups ranging from those that are psychologically close and smaller (family, friends, neighbors) to those that are more distal and larger (people living in Germany, humankind). We hypothesized that psychologically closer groups would be perceived as less affected by COVID-19 as well as less threatening than more psychologically distal groups. Based on social identity theorizing, we also hypothesized that stronger identification with humankind would change these patterns. Furthermore, we explored how these threat perceptions relate to adherence to COVID-19 health guidelines. In line with our hypotheses, latent random-slope modelling revealed that psychologically distal and larger groups were perceived as more affected by COVID-19 and as more threatening than psychologically closer and smaller groups. Including identification with humankind as a predictor into the threat target model resulted in a steeper increase in threat target perception patterns, whereas identification with humankind did not predict differences in threat source perceptions. Additionally, an increase in threat source perceptions across social groups was associated with more adherence to health guidelines, whereas an increase in threat target perceptions was not. We fully replicated these findings in a subgroup from the original sample (*N* = 989) four weeks later. We argue that societal recovery from this and other crises will be supported by an inclusive approach informed by a sense of our common identity as human beings.

The COVID-19 pandemic has been the most disruptive event of our lifetimes and in its year alone led to the loss of 2.6 million lives around the world (World Health Organization, 2021). In order to slow the spread of infection, individuals have had to adapt their behavior to follow health regulations and guidelines set out by health officials and governments. Adhering to these not only protects the individual but also supports the health and recovery process in society as a whole (Yzerbyt & Phalet, 2020). By the same token, underestimating risk may lead people to engage in behavior that is detrimental to health — for example, by having close contact with others when physical distancing is recommended (Cruwys et al., 2021).

As threat perceptions play a vital role in the motivation to engage in and sustain health-promoting behavior (Ferrer & Klein, 2015), it is important to understand how the threat posed by the pandemic is perceived as well as the factors that shape these perceptions. The contagious nature of COVID-19 increases the likelihood that social groups are perceived as both a *threat target* and a *threat source* (Greenaway & Cruwys, 2019). Accordingly, in the present research we investigate the perceived target *and* source of the threat posed by COVID-19 with respect to social groups that differ in terms of both their psychological distance and inclusivity/size. Indeed, because inclusivity and group size are correlated (such that larger, and more psychologically distal, groups are more inclusive), in what follows, we use these terms interchangeably. The groups that we examined as threat targets and threat sources were a) family/friends, b) neighborhood, c) nation, and d) humankind. We chose these particular groups because they were all observed to play an important role in the first phase of the pandemic (e.g., see Jetten et al., 2020). For example, in many cases, people’s contact was restricted to close friends and family, they were unable to travel outside their local community, and their behavior was regulated by the policies of national governments (Bundesregierung, 22 March, 2020; 15 April, 2020).

We also examined how representations of these threat targets and threat sources would predict adherence to COVID-19 health guidelines. Finally, we investigated whether participants’ identification with humankind, as the largest, most inclusive and most psychologically distal group, would change the threat target and threat source patterns.

## Targets of Threat

When it comes to understanding perceptions of health threat, research on the *optimistic bias* suggests that people tend to underestimate their personal risk of getting ill relative to the risk faced by other (Harris & Middleton, 1994; Weinstein, 1989). Underestimating risk also lowers people’s motivation to reduce risky health behavior. For example, smokers who underestimate their objective risk of getting lung cancer report lower motivation to quit smoking (Dillard et al., 2006).

**Figure 1:**
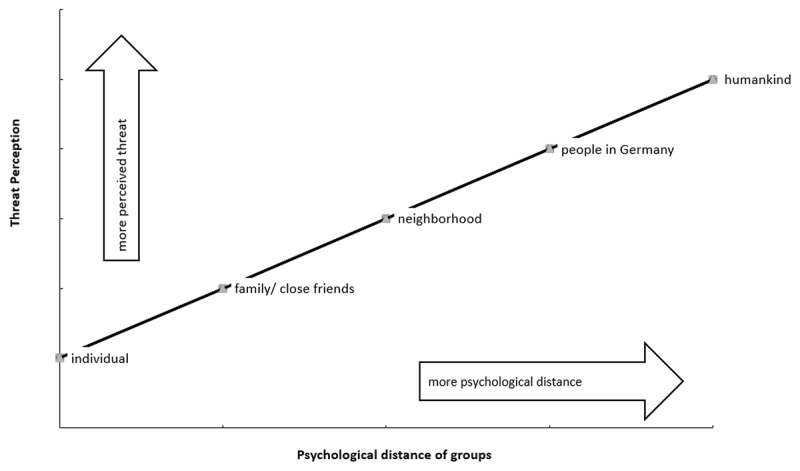
Simplified visualization of the threat target pattern (i.e., threat target slope).

However, the Social Identity Approach notes that people perceive themselves not only as individuals but also as members of social groups — with the social context determining which social identities they use as a basis for self-definition (Oakes et al., 1994; Turner et al., 1987). Here, other things being equal, the principle of perceiver readiness will often lead people to self-define as members of psychologically proximal groups with which they have more contact (e.g., their close family/friends or neighbors) rather than as members of more distal groups (e.g., their country or humankind; Oakes et al., 1994). As a result, the optimistic bias may extend to psychologically proximal groups but not to more distal ones. Moreover, if every group member displays an optimistic bias, a reciprocal strengthening of false immunity perceptions might occur within the group through a process of in-group projection (Wenzel et al., 2007) and/or group consensualization (Haslam et al., 1997). In-group projection involves seeing the group as a better (more prototypical) example of a superordinate social category than an out-group; group consensualization involves developing a collective consensus around a shared social identity. Together, these processes suggest that individuals may come to share the view that they and other members of psychologically proximal groups (e.g., family and close friends) are less at risk during the COVID-19 pandemic than more inclusive and psychologically distal groups (e.g., people in one’s own country or humankind). In particular, we assume that the perceived threat posed by the pandemic gradually increases in relation to the comparison category (that is: the individual) across family/close friends, neighbors, people in the country, and humankind. ***Figure 1*** illustrates the expected threat target pattern. Accordingly, we hypothesize that:

H1. Groups will be seen to be more of a *target* for COVID-19 infection the larger and more psychologically distal (vs. proximal) they are (i.e., there is a positive slope in threat target perceptions from more psychologically proximal to more distal groups).

## Sources of Threat

The perception that psychologically more distal groups are the (main) threat target may also strengthen the belief that, due to their higher risk of infection, these groups are also the main source of threat. Building on intergroup threat theory (Stephan et al., 2009; Stephan & Renfro, 2002), we thus propose that people will believe that they and their closely related social group members are more under threat when they come into contact with psychologically more distal and larger groups. Yet, because people are affected (and potentially harmed) by the behavior (i.e., (non-)adherence to Covid-19 guidelines) of psychologically more distal and inclusive groups during the pandemic, they may see themselves and psychologically proximal and exclusive groups as relatively powerless. To reduce this risk and gain a sense of control in this situation, they may therefore seek to reduce contact with ingroups that are psychologically more distal. Indeed, under these circumstances these groups may be recategorized as outgroups (Gaertner et al., 1989).

In line with our assumptions, Schlueter and Scheepers (2010) showed that relatively more inclusive (larger) groups are generally perceived as more threatening than more exclusive (smaller) ones. Accordingly, people should perceive themselves to be more at risk of infection from a member of a psychologically distal and larger group (e.g., an inhabitant of one’s country) than from a member of a proximal and smaller group (e.g., one’s family/friends). Furthermore, and in line with Social Identity Theory, people desire to achieve positive group distinctiveness (Tajfel & Turner, 1979), which may also lead people to downplay the threat posed by close and proximal ingroups (e.g., family and close friends) (Greenaway & Cruwys, 2019). In particular, the perceived threat posed by neighbors, people in one’s own country and people from other countries should gradually increase across these respective groups (relative to the closest group that is family/close friends). On this basis, we therefore hypothesize:

H2. Groups are more likely to be seen as a *source* of threat the larger and more psychologically distal (vs. proximal) they are (i.e., there is a positive slope in threat source perceptions from psychologically proximal to more distal groups).

## Consequences of Threat Perception Biases

Besides affecting perceptions of threat target and sources, social identity processes may also affect peoples’ willingness to adhere to COVID-19 health guidelines. In particular, if people underestimate the risk faced by themselves and psychologically proximal ingroups (i.e., they show a threat target bias), this may reduce their adherence to health guidelines. For instance, they might be less likely to socially distance themselves from others. In contrast, if people perceive there to be a stronger threat posed by inclusive and psychologically distal groups, they should be more willing to adhere to guidelines in order to protect themselves and close others. In line with this reasoning, there is initial evidence that perceptions of vulnerability (González-Castro et al., 2021) and realistic threat (Kachanoff et al., 2021) have been related to more adherence to health guidelines during the pandemic. Likewise, feeling more threatened has been found to be positively related to adherence (Reinders Folmer et al., 2020). Accordingly, we hypothesize:

H3. People who report stronger threat target biases (i.e., a steeper increase of the threat target slope across the groups) report less adherence to COVID-19 health guidelines.

H4. People who report stronger threat source biases (i.e., a steeper increase of the threat source slope across the groups) report more adherence to COVID-19 health guidelines.

## Reducing Threat Perception Biases

To reduce behavior that is detrimental to health, such as non-compliance with relevant guidelines, it is important to investigate factors that might lead people to rate threats more realistically. This means in particular that the threat posed by the pandemic also affects proximal groups and these groups can, in turn, be a source of threat. Here, social identity theorizing leads us to anticipate that individuals are more likely to see a given threat as a threat to ‘us’ (and hence as something to be taken seriously), the more they define themselves as members of the group in question (Haslam et al., 2018; van Dick & Haslam, 2012). Thereby, such social identification processes should reduce group differences on the respective threat dimensions (i.e., threat target and threat source) and at the same time, similarities, such as a common fate of being in the pandemic that bind people together, step into focus. Consequently, identification with a more inclusive group should affect people’s perceptions of threat targets and threat sources.

In particular, we chose identification with humankind, the most inclusive group, because such symbolic group memberships (i.e., identifying with abstract and psychologically distal groups) function as “stable anchors” during demanding and insecure times in ways that have positive consequences for social integration (Khan et al., 2014; 2020). Furthermore, the COVID-19 pandemic has been a global health crisis that has affected people worldwide rather than only those in only a specific country or community. Accordingly, a sense that ‘we are all in this together’ as when internalizing a more inclusive social identity should increase the awareness of a common threat in such a way that individuals differentiate to a lesser degree between threat posed by and for differently sized groups. Through this, the tendency to ‘outsource’ the threat source to more psychologically distal groups will be reduced. More specifically, we hypothesize:

H5. The more a person identifies with humankind the more the perceived threat for the groups increases (i.e., there is a positive association between identification with humankind and the positive slope of the threat target perception).

H6. The more a person identifies with humankind the less the perceived threat posed by the groups increases (i.e., there is a negative association between identification with humankind and the positive slope of the threat source perception).

## Method

### Sample and Procedure

The study was conducted in Germany as part of a multi-national research project. Ethical approval was obtained from the Faculty of Commerce’s Ethics Board (University of Cape Town; REF: REC 2020/03/013). We aimed to recruit a large and heterogeneous sample in Germany. As the pandemic itself affected the data collection process (e.g., because people were in lockdown or died) and to accelerate the data collection process we recruited our sample online via the panel provider Kantar.^1^ At the end of the first survey (T1: 26 March – 31 March 2020), respondents were asked if they would agree to participate in a follow-up survey about four weeks later (T2: 27 April – 4 May 2020). To test the generalizability of our findings and ensure that our findings were not restricted to just one short study period (i.e., T1), we tested our hypotheses at both measurement points.

Overall, 1623 individuals clicked on the link to the first study survey, 1502 started answering the questionnaire, and 1484 answered all questions. However, seven people participated more than once, thus, we only used their initial responses at T1, leaving us with a sample of *N* = 1475. Of these 1475 participants, 1015 also participated at T2 (five participated more than once but again we only counted their initial response). This resulted in a sample of *N* = 1010 at T2. In addition, we excluded the responses of two individuals at T1 and T2, who did not live in Germany.

Prior to determining the final sample, we checked the response quality by analyzing the meta-data of T1 and T2 based on the guidelines by Buchanan and Scofield (2018). Buchanan and Scofield (2018) recommended excluding participants not on the basis of a single indicator (e.g., response time), but on the basis of a combination of indicators. Therefore, we inspected participants’ answers to an open-ended question (flagging those with clearly insincere responses, such as using random letters), page response times (flagging those whose response time was less than 50% of the calculated median of the average response time of the sample; see Kaluza et al., 2021 for a similar approach), and inspected uniformity of answers across questionnaires in combination with the number of answer options used (flagging those who gave the same response to all questions). We then excluded participants who were flagged on at least two of the three indicators. After excluding 19 participants at T1 and 19 participants at T2 due to questionable data quality, the final sample was comprised of 1454 participants at T1 and 989 at T2. Their average age was 47.85 years (T1: *SD* = 15.15, range from 18-87; T2: *M* = 51.00, *SD* = 13.82, range from 18 to 87); 53.9% were women (T2: 52.8% women).

At the time the study was conducted 64.0% of the sample indicated being employed (T2: 59.8%) and 53.4% had at least one child (T2: 56.9%).^2^ At both measurement points some participants preferred not to answer questions about their current COVID-19 circumstances. Nevertheless, very few had tested positive for COVID-19 (T1: 1.2%, two missing values; T2: 0.3%, five missing values). Nearly a third of the respondents (T1: 28.7%, six missing values; T2: 24.5%, one missing value) took care of individuals who belong to high-risk groups (e.g., older adults), and almost half of the sample lived together with or close by members of high-risk groups (T1: 42.7%, six missing values; T2: 40.5%, one missing value). Only a few participants (T1: 4.3%, one missing value; T2: 3.7%, one missing value) indicated that a family member or close friend had tested positive for COVID-19. At T1, 13.1% lived in a high-risk region (T1: nine missing values; T2: 12.0%, three missing values).

### Measures

As this study was part of a larger research project, further measures were included in the study. Only measures relevant to this paper are reported here.

#### Perceptions of Threat Target

Participants rated the perceived threat posed by COVID-19 to themselves and four different social groups (‘At the moment, the threat for [me; my family/close friends; my neighbors; my country; humankind] is…’) on a scale from *1 = very low* to *5 = very high*.

#### Perception of Threat Source

Participants rated the threat faced by four different social groups (‘During the Coronavirus outbreak, to what extent do you feel threatened by [your family/close friends; your neighbors; people in your country; people from other countries]?’) on a scale from *1 = not threatened at all* to *5 = very threatened*.

#### Adherence to Health Guidelines

We developed eight items to measure adherence to health guidelines during the pandemic based on the recommendations that were in place in Germany at the onset of the pandemic (March/April 2020). We asked participants to indicate on a scale from *1 = strongly disagree* to *5 = strongly agree* how much they would agree with each of the following statements: (a) ‘I try to reduce social contacts to the bare minimum’; (b) ‘I keep away from public places’; (c) ‘I make sure to keep a distance of at least 1.5 meters (5 feet) between myself and other people’; (d) ‘I don’t meet with friends physically anymore’; (e) ‘I try to protect high-risk individuals (e.g., elders, people with chronic diseases) by keeping my distance from them’; (f) ‘I support high-risk individuals (e.g., elders, people with chronic diseases), for instance, by offering them help with shopping’; (g) ‘I encourage others to follow the recommendations to keep a distance, washing hands etc.’; (h) ‘I offer emotional support to members of my family (e.g., calling my parents/grandparents more frequently than usual’ (α_T1_ = .80, α_T__2_ = .79).

#### Identification with Humankind

Participants responded on a scale from *1 = strongly disagree* to *5 = strongly agree* to four items adapted from Doosje et al. (1995) to operationalize identification with humankind (‘I identify myself with other humans.’; ‘I am a part of humankind.’; ‘I feel strong ties with humankind.’; ‘I am glad to be part of humankind.’; α_T1_ = .90, α_T2_ = .90).

### Analysis

Data was processed with both SPSS v.26 (IBM Corp., 2020) and Mplus v.8 (Muthén & Muthén, 1998–2011). First, basic descriptive statistics and correlations for both samples (T1 and T2) were calculated to explore the distribution and relationships within the data. Second, and to test H1 and H2, the progressive trajectory or ‘sequential distance’ between threat target perception and threat source perception was assessed using structural equation modelling and by determining the robust maximum likelihood estimator (MLR). Two latent factors were estimated for each variable: (a) an *intercept* (with loadings constrained to 1) that reflected the average perception of threat target or threat source, and (b) a *linear slope* (constrained to either increments of 0, 1, 2, 3, 4 or left partially unconstrained) which reflected the change in relative distance between threat perceptions (Wang & Wang, 2020). For both variables, the fully constrained models assumed that the slope was comprised of equal perceptual distances between factors, represented by increments of 1. For the partially unconstrained models, two separate approaches were employed. For threat target perceptions, we used a partially unconstrained model where, the slope of ‘threat for me’ was constrained to be 0, the slope of ‘threat for my family/close friends’ was constrained to 1, and all other indicators were freely estimated. For source threat perceptions, the slope of ‘threat by my family/close friends’ was constrained to 0, the slope of ‘threat by my neighborhood’ was constrained to 1, and all other indicators were freely estimated. The intercept and slopes for all models were allowed to covary. Model fit was evaluated by the conventional methods recommended by Wang and Wang (2020).

To test H3 and H4, we examined whether the slopes of both the threat target and threat source models predicted adherence to health guidelines, which was estimated as a single factor latent variable. To test H5 and H6, we tested whether identification with humankind, which was estimated as a single-factor latent variable, predicted the slopes of both the threat target and threat source models. No additional constraints or modifications were permitted. Model fit was estimated using the same fit statistics, and statistical significance for the relationship was set at *p* < .05. As noted above, analyses were performed in both samples to test whether the observed pattern of support for our hypotheses would replicate over time.

## Results

***Table 1*** summarizes the descriptive statistics at both measurement points; ***Table 2*** presents the Pearson correlations for study variables at T1 and T2. In what follows, we first present the results of the analysis of data from the sample at T1 and then follow this with the results of the analysis of data from the T2 sample.

**Table 1:** Means (*M*) and Standard Deviations (*SD*) for N = 1454 (T1) and N = 989 (T2).


	T1			T2	
	
	*M*	*SD*		*M*	*SD*

1 Threat for the individual	3.11	1.08		2.86	1.06

2 Threat for family/close friends	3.29	0.99		2.99	1.02

3 Threat for neighborhood	3.20	0.92		2.99	0.92

4 Threat for country members	3.80	0.82		3.36	0.91

5 Threat for humankind	4.06	0.87		3.71	0.96

6 Threat by family/close friends	2.12	1.17		2.00	1.13

7 Threat by neighborhood	2.31	1.18		2.21	1.14

8 Threat by country members	3.07	1.19		2.81	1.15

9 Threat by people from different countries	2.89	1.32		2.60	1.24

10 Identification with humankind	3.88	0.83		3.82	0.85

11 Adherence to Covid-19 health guidelines	4.15	0.65		3.95	0.66


**Table 2:** Correlations for *N* = 1454 (T1) and *N* = 989 (T2).


	1	2	3	4	5	6	7	8	9	10	11	12	13	14	15

1 Gender^1^		.15***	–.06	.00	–.00	–.01	.03	–.03	.01	.02	–.01	–.06	–.04	–.03	–.16***

2 Age	.15***		.33***	–.37***	.11***	.02	.07*	.06	.10***	–.12***	–.09**	–.04	–.05	.17***	.06*

3 Children^2^	–.05	.36***		–.10**	.06	.01	.09**	.05	.07*	–.01	.02	.04	.07*	.11***	.12***

4 Job^2^	–.02	–.34***	–.08**		–.04	–.03	–.02	–.06	–.08*	.03	–.02	.00	.02	–.06	.03

5 Threat for the individual	–.06*	.09***	.09***	–.03		.81**	.70***	.59***	.46***	.41***	.41***	.48***	.34***	.11***	.34***

6 Threat for family/close friends	–.12***	–.03	.04	–.06*	.74***		.74***	.63***	.51***	.41***	.42***	.48***	.32***	.13***	.37***

7 Threat for neighborhood	–.05	.03	.06*	–.04	.69***	.75***		.66***	.51***	.33***	.44***	.45***	.31***	.17***	.37***

8 Threat for country members	–.08**	.08**	.09**	–.03	.47***	.50***	.50***		.77***	.24***	.34***	.48***	.33***	.16***	.40***

9 Threat for humankind	–.09**	.12***	.08**	–.03	.36***	.37***	.38***	.75***		.17***	.25***	.43***	.29***	.23***	.40***

10 Threat by family/close friends	.03	–.14***	.02	.05	.39***	.36***	.35***	.17***	.09***		.65***	.48***	.43***	.00	.13***

11 Threat by neighborhood	.03	–.11***	.02	.02	.40***	.36***	.43***	.20***	.12***	.65***		.66***	.50***	.00	.19***

12 Threat by country members	–.04	–.09***	.03	.01	.39***	.39***	.39***	.38***	.32***	.46***	.59***		.63***	.04	.31***

13 Threat by people from different countries	–.00	–.04	.06*	.02	.31***	.29***	.34***	.29***	.25***	.41***	.52***	.67***		.02	.17***

14 Identification with humankind	–.03	.11***	.09***	–.04	.04	.09***	.07**	.15***	.19***	–.06*	–.04	–.06*	–.10***		.30***

15 Adherence to Covid–19 health guidelines	–.18***	.03	.06*	.05	.23***	.27***	.23***	.32***	.31***	.03	.10***	.20***	.15***	.33***	


*Note*: * = *p* < .05, ** = *p* < .01, *** = *p* < .001. Correlational coefficients for study 1 (T1) under and for study 2 (T2) over the diagonal. ^1^ woman = 0, man = 1, other = 2. ^2^ no = 0, yes = 1.

### Dropout Analyses

Participants who only participated in the first survey (*M* = 41.08, *SD* = 15.69) were significantly younger than participants who participated in both surveys (*M* = 51.00, *SD* = 13.82), *t* (817.93) = –11.69, *p* < .001. However, there were no gender differences between participants who only participated in the first survey and those who participated in both surveys (χ^2^ (1) = 1.51, *p* = .22).^3^

### T1 Sample

#### Tests of H1: Perceptions of Threat Target

Analysis indicated that the partially unconstrained model fitted the data better than the intercept-only or constrained models (***Table 3***). In what follows, we report the results for the unstandardized model.

**Table 3:** Competing Trajectory Models at T1.


MODEL	*X* ^2^	*DF*	*P* VALUE	TLI	CFI	RMSEA	SRMR	90% C.I RMSEA		AIC	BIC	SCALING CORRECTION	MODEL COMPARISON	*SB*-Δ*X*^2^	Δ*DF*

								LL	UL						

*Threat Target*															

Model 1a: Intercept Only	2596.17	10	<.001	0.00	0.00	0.42	0.41	0.408	0.435	19596.21	19649.04	1.6157	Model 1a vs. Model 2a	not identified	0

Model 2a: Fully Constrained	624.52	10	<.001	0.76	0.76	0.21	0.08	0.192	0.219	16197.86	16250.68	1.2748	Model 2a vs. Model 3a	418.06*	3

Model 3a: Partially Constrained	74.18	7	<.001	0.97	0.96	0.08	0.05	0.065	0.098	15488.39	15557.05	1.0877	Model 3a vs. Model 1a	1444.66*	3

*Threat Source*															

Model 1b: Intercept Only	1559.19	6	<.001	0.00	0.00	0.42	0.37	0.404	0.440	18751.12	18793.38	1.5359	Model 1b vs. Model 2b	495.99*	1

Model 2b: Fully Constrained	402.24	5	<.001	0.74	0.69	0.23	0.13	0.215	0.253	16779.63	16827.17	1.0473	Model 2b vs. Model 3b	317.09*	2

Model 3b: Partially Constrained	16.24	3	.001	0.99	0.98	0.06	0.03	0.031	0.083	16376.82	16434.92	0.8902	Model 3b vs. Model 1b	1091.08*	3


*X*^2^ = Chi-square; *df* = degrees of freedom; TLI = Tucker-Lewis Index; CFI = Comparative Fit Index; RMSEA = Root Mean Square Error of Approximation; SRMR = Standardised Root Mean Square Residual; AIC = Akaike Information Criterion; BIC = Bayes Information Criterion; LL = Lower Level; UL = Upper Level; * statistically significant (*p* < 0.05); SB-ΔX^2^ Satorra-Bentler Scaled Chi Square Diff test.

The intercept of the partially unconstrained linear model, which indicates the average perception of threat target across the different targets, was 3.13 (*SE* =.03, *z* = 114.44, *p* < .001) and the estimated slope was .11 (*SE* = .02, *z* = 5.77, *p* < .001). In line with H1, this positive slope indicates that perceptions of threat target increased linearly as the distance increased from threat for the individual to threat for the individual’s family/close friends, neighborhood, country, and humankind. Further, the variances for both the intercept (σ*^2^* = .77, *SE* = .04, *z* = 22.05, *p* < .001) and the slope (σ*^2^* = .01, *SE* = .00, *z* = 3.01, *p* = .003) were significant — indicating that threat perceptions posed by COVID-19 differed as a function of its (potential) target. Moreover, the covariation between the intercept and the slope was significant (γ = –.06; *SE* = .01, *z* = –5.26, *p* < .001), indicating that respondents who started at a higher threat target level had a weaker increase (i.e., flatter slope) in threat target perceptions. In other words, those people differentiated less in their threat target perceptions towards different groups.

#### Tests of H2: Perceptions of Threat Source

The analysis for perception of the threat posed by different sources showed that the partially unconstrained model fitted the data better than the intercept-only model or the constrained linear model (***Table 3***). In the partially unconstrained linear model, the average level of threat source perception (i.e., the intercept) was 2.11 (*SE* = .03, *z* = 69.16, *p* < .001). Supporting H2, the estimated mean value for the slope was .22 (*SE* = .03, *z* = 8.09, *p* < .001), reflecting the fact that there was a progressive linear increase in the perceived threat posed by family/friends, the neighborhood, people of one’s own country, and people from other countries, respectively. Finally, the variances in both the intercept (σ*^2^* = .95, *SE* = .05, *z* = 20.17, *p* < .001) and slope (σ*^2^* = .04, *SE* = .01, *z* = 3.82, *p* < .001) were significant, indicating that individuals varied in their overall perceptions of threat source across ratings of psychologically proximal to more distal social groups. As with perceptions of the threat target, the covariation of the intercept and the slope of threat source perception was also significant (γ = –.07; *SE* = .01, *z* = –4.95, *p* < .001). This indicates that respondents who started out with higher threat source levels showed weaker increases (i.e., flatter slope) in threat source perceptions across the respective groups.

#### Tests of H3 and H4: Predicting Adherence to Health Guidelines

The model for threat target perception, in which the slope observed above (under H1) predicted adherence to health guidelines, had a poor fit to the data (*X*^2^(11) = 251.70, *p* < .001, scaling correction factor for MLR = 1.12, RMSEA = .12 [90% CI: .110, .136], CFI = .92, TLI = .89, SRMR = .13). Counter to H3, being more likely to see psychologically distal groups as a threat target was unrelated to adherence to health guidelines (γ = .18; *SE* = .21, *z* = .88, *p* = .380). This implies that people who perceived psychologically distal groups to be more affected by the pandemic relative to themselves did not adhere less to health guidelines (as predicted under H3).

However, the model for threat source perception, in which the slope observed above (under H2) predicted adherence to health guidelines, had a good fit to the data (*X*^2^(6) = 42.57, *p* < .001, scaling correction factor for MLR = 0.87, RMSEA = .07 [90% CI: .047, .084], CFI = .98, TLI = .97, SRMR = .05). In line with H4, being more likely to see psychologically distal groups as a source of threat predicted greater adherence to health guidelines (γ = .70; *SE* = .14, *z* = 5.06, *p* < .001). In other words, the more individuals perceived other groups as more threatening relative to their families/close friends, the more they adhered to health guidelines.

#### Tests of H5 and H6: Identification with Humankind Predicts Threat Target and Threat Source Patterns

The model for threat target perception, in which identification with humankind was included as a predictor of the slope observed above (under H1), had a good fit to the data (*X*^2^(10) = 80.04, *p* < .001, scaling correction factor for MLR = 1.08, RMSEA = .07 [90% CI: .056, .084], CFI = .98, TLI = .96, SRMR = .04). In line with H5, identification with humankind positively predicted the slope of perceptions of threat targets (γ = .02; *SE* = .01, *z* = 3.08, *p* = .002). Thus, the more individuals identified with humankind, the steeper the increase in threat target perception across the respective target groups.

The model for threat source perception, in which identification with humankind was included as a predictor of the slope, likewise had a good fit to the data (*X*^2^(5) = 23.17, *p* < .001, scaling correction factor = 0.95, RMSEA = .05 [90% CI: .031, .071], CFI = .99, TLI = .98, SRMR = .03). However, the association between identification with humankind and the slope of threat source perception was not significant (γ = –.01; *SE* = .01, *z* = –.61, *p* = .55). Accordingly, H6 was not supported. In other words, even when people identified with humankind, they still tended to ‘outsource’ potential threat sources to more psychologically distal groups.

### Time 2 Sample

#### Tests of H1: Perceptions of Threat Target

As for the T1 sample, the partially unconstrained model for threat target perception fitted the data better than the intercept-only model or the constrained linear model (***Table 4***). In this model, the average level of threat target perception (i.e., the intercept) was 2.86 (*SE* = .03, *z* = 87.30, *p* < .001). The estimated mean value for the slope was .09 (*SE* = .02, *z* = 5.24, *p* < .001), which implies a linear increase in threat target perception as the distance increased from threat for the individual to threat for the individual’s family/close friends, neighborhood, country, and humankind, supporting H1 and replicating the results from T1. In line with the results in the T1 sample, the variances in intercept (σ*^2^* = .83, *SE* = .04, *z* = 19.95, *p* < .001) and slope (σ*^2^* = .01, *SE* = .00, *z* = 2.73, *p* = .006) were significant, which implies that individuals varied significantly in their overall perceptions of threat depending on the potential target group. Again, the covariation of the intercept and the slope of the threat target perception were significant (γ = –.04; *SE* = .01, *z* = –4.59, *p* < .001), indicating that individuals who started at a higher threat target level showed a weaker increase in perceived threat the more distal the group that was being judged. In other words, these people differentiated less in heir threat target perceptions towards the different groups.

**Table 4:** Competing Trajectory Models at T2.


MODEL	*X* ^2^	*DF*	*P* VALUE	TLI	CFI	RMSEA	SRMR	90% C.I RMSEA		AIC	BIC	SCALING CORRECTION	MODEL COMPARISON	SB–Δ*X*^2^	Δ*DF*

								LL	UL						

*Threat Target*															

Model 1a: Intercept Only	1918.24	10	<.001	.00	.00	.44	.46	.423	.456	13766.98	13815.94	1.8083	Model 1a vs. Model 2a	not identified	0

Model 2a: Fully Constrained	262.43	10	<.001	.87	.87	.16	.07	.143	.177	10626.44	10675.41	1.2507	Model 2a vs. Model 3a	126.23*	3

Model 3a: Partially Constrained	88.48	7	<.001	.96	.94	.11	.06	.089	.129	10401.31	10464.97	1.0974	Model 3a vs. Model 1a	971.73*	3

*Threat Source*															

Model 1b: Intercept Only	956.18	6	<.001	.00	.00	.40	.37	.379	.422	12426.21	12465.39	1.7218	Model 1b vs. Model 2b	298.51*	1

Model 2b: Fully Constrained	245.24	5	<.001	.75	.70	.22	.10	.197	.244	11061.54	11105.61	1.1404	Model 2b vs. Model 3b	162.65*	2

Model 3b: Partially Constrained	33.93	3	<.001	.97	.94	.10	.06	.073	.134	10815.59	10869.45	0.8762	Model 3b vs. Model 1b	629.67*	3


*X^2^* = Chi-square; *df* = degrees of freedom; TLI = Tucker-Lewis Index; CFI = Comparative Fit Index; RMSEA = Root Mean Square Error of Approximation; SRMR = Standardised Root Mean Square Residual; AIC = Akaike Information Criterion; BIC = Bayes Information Criterion; LL = Lower Level; UL = Upper Level; * statistically significant (*p* < 0.05); SB–ΔX^2^ Satorra-Bentler Scaled Chi Square Diff test.

#### Tests of H2: Perceptions of Threat Source

Consistent with the T1 results, the partially unconstrained model for threat source perceptions fitted the data better than the intercept-only model or the constrained linear model (***Table 4***). In this model, the average level of threat source perception was 1.98 (*SE* = .04, *z* = 56.30, *p* < .001). The estimated mean value for the slope was .25 (*SE* = .03, *z* = 7.95, *p* < .001). Supporting H2, there was again a progressive linear increase in the perceived threat posed by family/friends, the neighborhood, people from one’s own country, and people from other countries. Furthermore and again consistent with the T1 results, the variances in both intercept (σ*^2^* = .88, *SE* = .05, *z* = 16.34, *p* < .001) and slope (σ*^2^* = .07, *SE* = .02, *z* = 3.25, *p* = .001) were significant – implying that respondents varied in their overall perception of the threat posed by social groups that differed in their psychological proximity. Again, the covariation of the intercept and the slope of the threat source perception was significant (γ = –.08; *SE* = .02, *z* = –3.91, *p* < .001) indicating that respondents who started out with higher threat source levels showed weaker increases in their threat source perceptions as they judged more distal groups.

#### Tests of H3 and H4: Predicting Adherence to Health Guidelines

The T2 model for threat target perception, in which the slope observed above (under H1) predicted adherence to health guidelines, had a poor fit to the data (*X*^2^(11) = 303.78, *p* < .001, scaling correction factor for MLR = 1.07, RMSEA = .16 [90% CI: .148, .180], CFI = .88, TLI = .83, SRMR = .17). Consistent with the results of T1 and contrary to H3, being more likely to see distal groups as a threat target did not predict weaker adherence to health guidelines (γ = .18; *SE* = .30, *z* = .62, *p* = .536). This implies that people did not adhere less to health guidelines the more they outsourced the threat (i.e., perceived psychologically distal groups to be more affected by the pandemic relative to themselves).

The T2 model for threat source perception, in which the slope observed above (under H2) predicted adherence to health guidelines, had an acceptable fit to the data (*X*^2^(6) = 87.08, *p* < .001, scaling correction factor for MLR = 0.90, RMSEA = .12 [90% CI: .096, .139], CFI = .93, TLI = .90, SRMR = .09). In line with the results of T1 and supporting H4, the slope was positively related to adherence with health guidelines (γ = .69; *SE* = .16, *z* = 4.35, *p* < .001). This means that people adhered more to health guidelines when they had higher tendencies to perceive psychologically distal groups as a threat source.

#### Tests of H5 and H6: Identification with Humankind Predicts Threat Target and Threat Source Patterns

Consistent with the results from T1, the model for threat target perception, in which identification with humankind was included as a predictor of the slope, had a good fit to the data (*X*^2^(10) = 97.71, *p* < .001, scaling correction factor for MLR = 1.10, RMSEA = .09 [90% CI: .078, .112], CFI = .96, TLI = .94, SRMR = .06). In line with H5 and replicating the results obtained with the T1 sample, identification with humankind was positively related to the slope of threat target perception (γ = .01; *SE* = .01, *z* = 2.49, *p* = .013). Thus, the more individuals identified with humankind, the steeper the increase in threat target perception across the target groups.

Finally, and again consistent with the results based on the T1 sample, the model to test whether identification with humankind predicted the slope in the threat source model had a good fit to the data (*X*^2^(5) = 30.60, *p* < .001, scaling correction factor for MLR = 0.99, RMSEA = .07 [90% CI: .049, .097], CFI = .98, TLI = .95, SRMR = .05). However, there was no support for H6 as the association between identification with humankind and the slope of threat source perception was again not significant (γ = .02; *SE* = .02, *z* = 1.26, *p* = .21). This means that even though people felt connected to humankind they still perceived psychologically distal groups as the main threat source (compared to more psychologically proximal groups).

In summary, analysis of T2 data fully replicated the patterns of support for our hypotheses observed in the T1 sample one month earlier.

## Discussion

The purpose of this study was to investigate which social groups were perceived as a *threat target* and as a *threat source* during the COVID-19 outbreak. These groups varied in psychological distance and size, ranging from family/close friends, and the local neighborhood to people from one’s own country (in this case Germany), up to humankind. Our findings showed that more psychologically distal and larger groups were perceived as more likely to be targeted by COVID-19 and to be a source of threat than closer and more exclusive social groups. We also found that the increase in perceived threat source perceptions across groups was positively related to adherence to health guidelines, while the increase in perceived threat target perceptions was unrelated to adherence. Finally, there was evidence that identification with humankind increased the gradient of threat target perceptions, but did not affect the same gradient for perceptions of threat source.

Overall, these results suggest that people not only underestimate their own vulnerability, but also that of closely related ingroups (e.g., family and friends). In this way, it appears that the optimistic bias extends beyond the personal self and includes others who are part of relatively exclusive ingroup categories. As noted above, this might occur through processes of social identity-based in-group projection (Wenzel et al., 2007) or group consensualization (Haslam et al., 1997) whereby members of psychologically proximal and exclusive groups reinforce the perception that they are more immune and healthy than more distal groups.

The finding that the participants felt more threatened by larger groups accords with intergroup threat theory (Stephan & Renfro, 2002) and with previous research on (inter-)group threat perceptions and group size (Schlueter & Scheepers, 2010). As participants are, objectively speaking, part of the larger and more threatening groups (e.g., German residents and humankind), our results show that intergroup threat theory also applies to groups that a person is a member of but may nevertheless distance themselves from (perhaps by recategorizing them as an outgroup; Gaertner et al., 1989). However, it is not possible to establish whether the mechanism here is the perceived power of psychologically distal and more inclusive groups (i.e., the fact that these groups have more impact on the spread of the coronavirus than smaller groups) or group size per se (i.e., the fact that larger groups are more perceived to be more threatening), or a combination of both factors. In addition, it might also be the case that more inclusive groups imply a lower sense of control (e.g., because the probability of people not complying to health guidelines is larger) and that this in turn makes them more threatening for individuals. In order to clarify these issues, future research needs to investigate this question further.

The fact that the increase in threat target perceptions across different groups was unrelated to adherence to health guidelines implies that people did not adhere less because they perceived other social groups to be more threatened than themselves. However, in line with H4, people adhered more to health guidelines the more they perceived distal social groups to be more threatening relative to their family and close friends. At first glance, these results paint a rather egoistic picture of human behavior in crisis, as they suggest people take precautions when they perceive their loved ones to be threatened by other social groups, but do not do so when more psychologically distal groups are under threat.

Nevertheless, another point that should be raised here is the difference in the operationalization of the threat target and threat source dimensions. Whereas the threat target items framed the threat coming from the pandemic itself (and targeting groups), the threat source items described the threat coming from these respective groups. It could be that people might feel rather overwhelmed and powerless when thinking about the pandemic as it represents an abstract and uncontrollable threat. However, when the threat is seen to be posed by social groups, people might feel more self-effective in reducing the threat for themselves and their loved ones by adhering to health guidelines as these kind of measures have been proven to be effective against infections (Matrajt & Leung, 2020). Accordingly, this would explain why we found a relation between adherent behavior and threat source perceptions, but not with threat target perceptions. In line with this explanation, Kachanoff et al. (2021) found that different kinds of threats (realistic vs. symbolic) can either support or undermine adherence to social distancing measures.

In line with social identity theorizing, the perception of oneself as part of humankind strengthened the awareness that everyone is at risk for infection, because also psychologically more proximal groups (i.e., neighbors) are targets of the pandemic. Yet, identification with humankind did not alter perceptions of threat sources. In other words, even though some people identified more strongly with humankind, they felt similarly more threatened by more inclusive (vs. less inclusive) groups (i.e., people from other countries). Based on Roccas et al’s. (2008) multidimensional group identification model, one explanation for this finding could be that we only operationalized what these researchers referred to as the ‘importance dimension’ of identification with humankind. This dimension focuses on the internalization of the group membership into the self-concept. However, the respondents in our study might have differed on the *deference* dimension of identification with humankind. Deference refers to the degree to which group members honor, revere, and submit to the group’s norms, symbols, and leaders. Accordingly, we suggest that patterns of threat target and threat source perceptions may be affected by different dimensions of identification with humankind. The importance dimension might be crucial in strengthening the threat target pattern, because it comprises the perception of ‘we are all in this together’, which highlights the fact, that everybody was affected by the threat posed by COVID-19. In turn, the deference dimension (which we did not operationalize) might be more relevant when it comes to changing the threat source patterns. Variation in behavior might induce feelings of uncontrollability and powerlessness, especially when they violate individuals’ norms, values and beliefs (e.g., ‘It is important to adhere to health guidelines’) through non-adherence. The result is that respondents might have felt a bond with humankind due to the common fate, but (still) perceived the threat to stem from psychologically distal and inclusive social groups. However, to clarify the meaning of these results, future research should explore the impact of different dimensions of group identification, because some dimensions might be more relevant to particular perceptions of group threat than others.

### Practical Implications

Our findings suggest that achieving a realistic estimation of the threat posed by COVID-19 (or similar events) requires people to have a self-concept grounded in membership of inclusive rather than exclusive social groups. Indeed, people were likely to appraise this threat as more severe the more they understood that all of humanity is part of the same ingroup and, hence, that everybody is under threat. As (Yzerbyt & Phalet, 2020) noted, in order for them to engage in health-promoting behaviors during a pandemic individuals have to be aware that these behaviors not only protect their *own* health but that of society as a whole (see also Jetten et al., 2020; van Bavel et al., 2020).

To further manage the spread of infection and foster adherence to COVID-19 related restrictions, it is imperative that a sense of collective identity is established and maintained. In particular, this can be promoted through *identity leadership* (Haslam et al., 2020; Steffens et al., 2014; van Dick et al., 2018) which fosters cross-national collaboration, fights group-based discrimination, and strengthens a collective sense that ‘we are all in this together’ (Dovidio et al., 2020; Haslam et al., 2021).

### Limitations

As we already pointed out, pandemics are not conducive to optimal research design. Our use of single-source data and a cross-sectional design means that we cannot draw causal conclusions from our findings. Furthermore, our use of a panel provider in order to accelerate the data collection created the risk of recruiting a biased sample, in which some subgroups (e.g., people who are adversely affected by the pandemic and those who lack the time or where not motivated to participate in research) are underrepresented. The items for measuring perceptions of threat were also not validated, but rather formulated to fit the study’s needs. And although the items were not intended to shape the self-categorization of participants, it is possible that some item formulations made particular in-group/out-group categorizations more salient (in ways suggested by Oakes et al., 1994). Additionally, we used slightly different wordings for measuring the threat target (i.e., my country) and threat source perception (i.e., people in my country) that may have influenced threat representations as they imply different levels of abstraction. In particular, compared to the first formulation, referring to ‘people in my country’ might have primed a more concrete representation in ways that made the threat more salient. Finally, as we noted, perceived physical distance might be a confounding variable as psychologically closer social groups are also those that are physically closest (family, close friends or neighbors). Nevertheless, participants rated their families/close friends as being less threatening than their neighbors, which implies that they distinguished between psychological and physical distance. Moreover, despite these limitations, the large sample size and its quasi-representative nature — as well as the fact that we could fully replicate our models over a one-month period — gives us some confidence in the robustness and generalizability of the patterns we identified.

## Conclusion

The results of the present study indicate that people tend to underestimate the threat that COVID-19 poses for themselves and psychologically proximal (vs. distal) social groups. Additionally, larger and psychologically more distal social groups are perceived to be a source of greater threat. Our results are a first indication of the importance of people’s social identities for their appreciation of threat in the context of the pandemic. As well as helping us to understand these processes in the present these insights are also important for building a safe and harmonious society in the future. This, we hope, will be one where a sense of our common humanity will allow us not only to understand the shared threats we face but also to address them by building bridges between us rather than walls.

## Footnotes

Data are available upon request from the first author.

Based on participants’ indications at T1.

One participant identified as diverse and was excluded from this analysis.
